# Impact of Palliative Care on Interhospital Transfers to the Intensive Care Unit

**DOI:** 10.2478/jccm-2022-0009

**Published:** 2022-05-12

**Authors:** Safanah Tabassum Siddiqui, Emily Xiao, Sonika Patel, Kiran Motwani, Keneil Shah, Xinyuan Ning, Kathryn S. Robinett

**Affiliations:** 1 University of Maryland School of Medicine, Baltimore, MD, USA; 2 University of Rochester School of Medicine and Dentistry, Rochester, NY, USA; 3 Icahn School of Medicine at Mount Sinai, New York, NY, USA; 4 The University of Chicago Pritzker School of Medicine, Chicago, IL, USA

**Keywords:** palliative care, interhospital transfer, community hospital, academic health center, quality improvement

## Abstract

Community hospitals will often transfer their most complex, critically ill patients to intensive care units (ICUs) of tertiary care centers for specialized, comprehensive care. This population of patients has high rates of morbidity and mortality. Palliative care involvement in critically ill patients has been demonstrated to reduce over-utilization of resources and hospital length of stays. We hypothesized that transfers from community hospitals had low rates of palliative care involvement and high utilization of ICU resources. In this single-center retrospective cohort study, 848 patients transferred from local community hospitals to the medical ICU (MICU) and cardiac care unit (CCU) at a tertiary care center between 2016-2018 were analyzed for patient disposition, length of stay, hospitalization cost, and time to palliative care consultation. Of the 848 patients, 484 (57.1%) expired, with 117 (13.8%) having expired within 48 hours of transfer. Palliative care consult was placed for 201 (23.7%) patients. Patients with palliative care consult were statistically more likely to be referred to hospice (p<0.001). Over two-thirds of palliative care consults were placed later than 5 days after transfer. Time to palliative care consult was positively correlated with length of hospitalization among MICU patients (r=0.79) and CCU patients (r=0.90). Time to palliative consult was also positively correlated with hospitalization cost among MICU patients (r=0.75) and CCU patients (r=0.86). These results indicate early palliative care consultation in this population may result in timely goals of care discussions and optimization of resources.

## Introduction

Academic health centers provide highly specialized and comprehensive care, serving as referral centers for the larger community in which they reside. Surrounding hospitals often transfer their most critically ill patients to academic health center intensive care units (ICU) for further management, under the assumption that the advanced resources of a tertiary care centers will ultimately improve outcomes. However, these patients have a high rate of in-hospital mortality and other adverse outcomes, attributed to medical complexity and severity of illness [1,2]. Compared to direct admissions, patients transferred from outside hospitals are more severely ill, have longer ICU and hospital lengths of stay, utilize more resources, and accrue more costs [1,3]. With finite resources, academic health centers need to identify patients that are most likely to benefit from transfer, and also recognize the limitations of healthcare.

Palliative care is increasingly recognized as a vital component of providing comprehensive care in the ICU setting, regardless of patient prognosis [4]. It provides an individualized and family-centered approach to caring for a patient with a serious illness, which includes identifying goals of care, targeting symptom relief, and providing medical treatment according to patient preferences to improve quality of life. The principles of palliative medicine address the holistic needs of the patient and are beneficial when partnered with treatments of curative or life-prolonging intent [5].

Involvement of palliative care in intensive care settings has been shown to decrease hospital and ICU length of stay without increases in ICU mortality.

These findings are attributed to earlier advanced care planning and a subsequent reduction in unwanted treatments in the ICU [4]. Palliative care involvement is also associated with improved quality and quantity of communication with family members, facilitating implementation of do-not-resuscitate orders, and timely withdrawal of life-sustaining treatments when no longer beneficial [6]. Early palliative care involvement is particularly associated with an increased rate of hospice referrals [7]; such measures reduce over-utilization of resources [8,9]

While numerous studies show high rates of mortality and higher costs associated with critically ill patients after interhospital transfer, none we found have examined the impact of palliative care involvement in this cohort. The goals of this retrospective review are to (1) quantify outside hospital transfers to a single institution’s medical intensive care unit (MICU) and cardiac critical care unit (CCU) that were eligible for hospice referral during hospitalization, (2) identify the rate and timing to palliative care consultation, (3) define the relationship of palliative care consultation with outcomes, disposition, and cost.

Transfers from a community hospital to an academic health center often occur under the presumption that a specialized or life-saving intervention will take place. Consequently, it is possible that expectations of advanced interventions or greater treatment opportunities act as barriers to approaching goals of care discussions and palliative care involvement. We hypothesized that patients transferred to the MICU and CCU of an academic health center have low rates of palliative care consultation, hospice discharges, and a high utilization of MICU and CCU resources.

## Materials and methods

This is a single-center, retrospective chart review of all patients transferred to the University of Maryland Medical Center (UMMC) MICU and CCU from outside hospitals between 2016 and 2018. Data extracted from the electronic medical record included 475 MICU patients and 373 CCU patients for the duration of their hospitalizations.

### Setting

Located in downtown Baltimore, UMMC is an academic tertiary care center with robust transplant programs, a high-volume cardiothoracic surgery program, an advanced interventional radiology department, a center of excellence in extracorporeal membrane oxygenation (ECMO), and a center of excellence in cancer treatment. Patients are accepted for transfer to UMMC when an intervention can be offered that is not available at the outside hospital. Palliative care is an interdisciplinary consultative service at UMMC encompassing physicians, nurses, pharmacists, and social workers. Patients at UMMC qualifying for hospice may be referred for home hospice or inpatient hospice at another institution.

### Demographics

See Table 1.

**Table 1:** Patient Demographic Characteristics

	MICU		CCU	
Total Patients	475		373	

Gender				
Male	265	55.79%	233	62.47%
Female	210	44.21%	140	37.53%

Age				
Range	19-87		19-93	
Mean	57.57		67.08	
Median	59		69	
Mode	56		66	
Standard Deviation	14.27		14.65	

Ethnicity				
American Indian	1	0.21%	1	0.27%
Asian	6	1.26%	4	1.07%
Black/African American	157	33.05%	108	28.95%
White	265	55.79%	240	64.34%
Other/Unknown	46	9.68%	20	5.36%

First Code Status on Record*				
Full Code	259	54.53%	219	58.71%
Do NOT Intubate, No CPR	52	10.95%	43	11.53%
Intubate, No CPR	72	15.16%	17	4.56%
Limited	20	4.21%	6	1.61%
Palliative and Supportive Care, No CPR	49	10.32%	9	2.41%
None documented	23	4.84%	79	21.18%

APDRG Severity Index				
Extreme	426	89.68%	233	62.47%
Major	39	8.21%	75	20.11%
Moderate	9	1.89%	49	13.14%
Minor	1	0.21%	16	4.29%
Mechanical Ventilation	349	73.47%	144	38.60%

*Code status definitions: Full code – perform full scope of cardiopulmonary resuscitation including intubation. Limited – perform limited specific resuscitation procedures as communicated by patient or health care proxy and documented. Palliative and supportive care – comfort care only.

### Measurements

Primary data endpoints collected were length of stay, hospitalization cost, disposition (deceased, hospice, other discharge), presence of palliative care consultation, and time to palliative care consultation. Additional data included All Patients Refined Diagnosis Related Groups (APRDRG) severity, primary diagnosis by ICD code, insurance type, and transferring hospital. Determination of appropriate APRDRG classification is disease-specific and reliant upon the presence and burden of other co-morbid conditions. These cases are further stratified into severity of illness subclasses and risk of mortality subclasses in concordance with standardized methodology by 3M [10].

### Statistical Analysis

Descriptive statistics were used to report in-hospital mortality rate, mortality rate during first 48 hours of hospitalization. Descriptive statistics were used to report length of stay and cost of hospitalization for all patients and for patients who died within 48 hours.

An independent t-test was conducted to compare mortality rate, percentage of hospice referrals, mean length of stay, and mean cost of hospitalization between patients with and without palliative care consultation. Pearson correlation coefficient was calculated to analyze relationship between time to palliative care consultation with length of hospitalization as well as palliative care consultation with length of stay.

## Results

Of the 848 patients, 484 (57.1%) died during hospitalization. Among those that died, 117 (13.8%) died within 48 hours of transfer. The APRDRG severity index categorized 67.6% of patients as extreme and 13.4% as major. 74.9% of the patients within the extreme category and 16.7% in the major category died. 349 (73.4%) of MICU patients and 144 (38.6%) of CCU patients required mechanical ventilation.

**Fig. 1. A:**
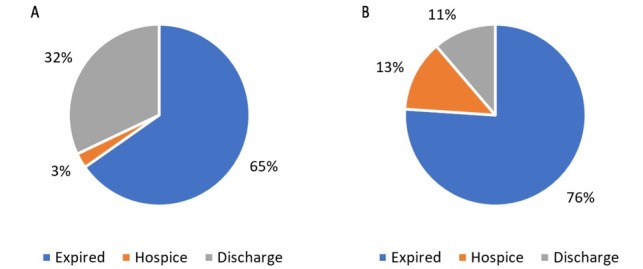
Disposition of patients without palliative care consult. B. Disposition of patients with palliative care consult.

Palliative care was consulted for 201 (23.7%) total patients, and 195 (26.7%) patients who survived beyond 48 hours. Of all the transfers, 49 (5.8%) were referred and discharged to hospice. Of patients referred to hospice, 34 (69.4%) had a palliative care consult (Figure 1A-B).

Of CCU patients, 16 (26.7%) with palliative care consults were referred to hospice and 5 (1.6%) without palliative care consults were referred to hospice (p<0.001). Of MICU patients, 18 (12.8%) with palliative care consults were referred to hospice and 10 (3.0%) without palliative care consults were referred to hospice (p<0.001).

Of all patients, 31% were black and 60% were white. Of patients with palliative care consults, 33% were black and 58% were white. Of those referred to hospice, 28% were black and 65% were white.

Mean length of stay was 15 days (range 0 to 205 days) for MICU patients and 8 days (range 0 to 90 days) for CCU patients. Mean length of stay for all transferred patients was 11.6 days. Mean length of stay for patients with palliative care consults was 20.6 days and 8.8 days for patients without palliative care consults (p<0.001). Average time to disposition after palliative care consult was 8.4 days, 5.3 days for patients who were discharged to hospice, and 6.9 days for patients who died.

Mean cost of hospitalization for all transferred patients was $62,548.38. Mean cost of hospitalization was $77,185.62 for MICU patients and $43,908.46 for CCU patients. Mean cost of hospitalization for patients with palliative care consults was $100,815.15 and for patients without palliative care consults was $50,660.25 (p<0.001).

**Fig. 2:**
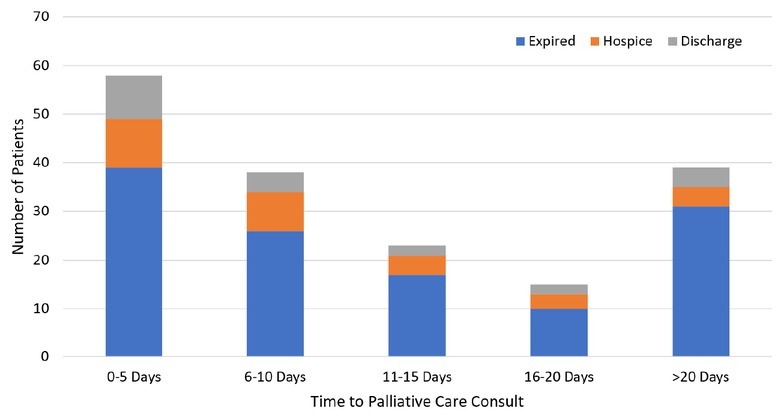
Distribution of time to palliative care consult and disposition (MICU and CCU)

**Fig. 3:**
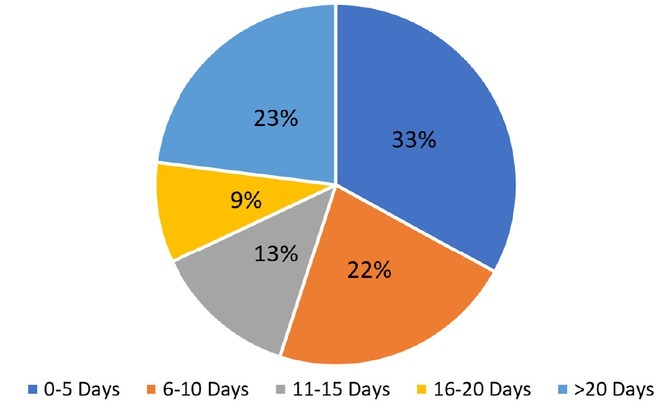
Time to palliative care consult

Mean time to palliative care consult was 8.6 days for CCU patients (range 0 to 52 days) and 13 days for MICU patients (range 0 to 116 days) (Figure 2). Of MICU patients with palliative care consults, 60% of consults were placed more than 7 days after admission (Figure 3).

**Fig. 4:**
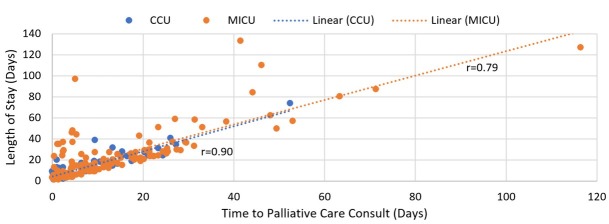
Time to palliative care consult and length of stay

Among MICU patients, time to palliative care consultation had a positive correlation with the length of hospitalization with r=0.79 and with hospitalization cost with r=0.75. Among CCU patients, time to palliative care consultation had a positive correlation with the length of hospitalization with r=0.90 and with hospitalization cost with r=0.86. (Figure 4)

Most common principal diagnoses for patients in the MICU were sepsis (40.4%), respiratory failure (11.8%), and liver failure (10.9%). Malignancies were listed as principal diagnoses for 9.3% of MICU patients.

Of the patients transferred to the CCU, the most common diagnoses for transfer to the CCU included acute myocardial infarction (MI) (28%), non-cardiac (22%), congestive heart failure (CHF) requiring inotropes or mechanical circulatory support (17%), and percutaneous valve replacement (14%). Of those patients with CHF, 60% died and 22% had palliative care consults. All CHF patients who went to hospice had palliative care consults. Of those 104 patients with MI, 35 (33%) died and only eight (7.7%) had palliative care evaluations.

## Discussion

Our findings confirmed the high mortality rate, costs, and lengths of stay associated with outside hospital transfers discussed in previous studies [1,2,3]. The low rate of consultation suggests underutilization of palliative care amongst patients transferred from outside hospitals. Patients with palliative care consults were significantly more likely to be referred to hospice, but still had high mortality rates, suggesting a missed potential to facilitate timely goals of care discussions.

Patients in whom palliative care was consulted had longer length of stays and higher hospitalization costs compared to those who did not have palliative care consult. It is possible that those with palliative care consultation represented a sicker overall cohort than the other group, represented by the 13.8% mortality rate within 48 hours of transfer. In addition, most palliative care consults were placed greater than 7 days after admission. Longer time to palliative care consult was significantly associated with longer hospitalizations and higher costs. Causation cannot be implied, so it cannot be determined if earlier palliative care consults would result in reduced costs and shorter hospital stays. Alternatively, longer hospitalization could have been a common trigger to palliative care consultation as a final effort after having exhausted aggressive interventions. This could be explained by physician attitude, lack of standardization, or poor measures of prognostication.

Delay in palliative care consultation may represent a perception of palliative care involvement as a “last resort” at the end of life, rather than an available resource to be employed preemptively. Multiple studies have demonstrated the benefits of palliative care in addressing the physical and psychological sequelae of surviving a stay in the ICU [11], including improving quality and quantity of communication with patients’ families throughout the hospital admission [6].Furthermore, recent literature has expanded upon the severity and scope of the “postintensive care syndrome” impacting both patients and families after hospital discharge. Among patients, physical debility, chronic fatigue and pain, and clinically significant depression are very prevalent months to years after ICU discharge. Among family members of critically-ill patients, anxiety, depression, post-traumatic stress disorder, and complicated grief also may persist. Proactive palliative care involvement during the acute critical illness could not only improve patient and family experience during the hospitalization but also better prepare them for the challenges faced in the months following discharge [12].

Data suggests that intensive care physicians are receptive to the use of palliative care in the ICU setting [13], although there is rarely a standardized approach to doing so [14]. Triggers to involve palliative care are rarely objective scoring systems, but rather a gestalt estimation of prognosis factoring in age, severity of illness, and subjective judgment of quality of life [14,15]. According to self-reports from ICU clinicians, length of hospital stay is not a common trigger [14].

Studies have shown that an integrative approach of palliative care with the ICU care team decreases ICU length of stay and improves resource utilization [16]. Integration could potentially eliminate the lag time to palliative care consultation and could possibly equip ICU teams with the ability to provide care from a palliative care model, without depending on the limited availability of palliative care specialists. An alternative model is a trigger-based model, where patients who meet certain criteria of severity of illness or underlying end-stage comorbidities in an ICU trigger a palliative care consultation, minimizing provider subjectivity or bias [17]. While these triggers have not been universally established, it is estimated that 14-20% of ICU admissions meet criteria for a trigger [18], though we do not have sufficient data to determine if this applies to our study population. This model has been associated with more frequent “do not resuscitate” code status orders and hospice referrals [19], and has been argued to improve patient comfort [17]. Our institution does not employ these models but has a traditional palliative care consultant team.

When stratified by diagnosis, we found only a small proportion of patients with CHF ended up receiving a palliative care consult, despite a very high mortality rate. It is unclear what is accounting for this disparity; however, it is possible that interfacility transfer for the initiation of advanced interventions may have served as a barrier for initiation of goals of care discussions with patients or their families. Prior studies have demonstrated that discharge hospice referrals to CHF patients lead to decreased readmission rates without changes to all-cause mortality [20]. Patients with end-stage CHF often experience debilitating dyspnea, pain, and anxiety, leading to multiple hospital readmissions prior to death [21]. The lower readmission rate among patients receiving discharge hospice referrals may suggest that the palliative approach used by hospice teams was effective in alleviating these symptoms and removing the need for hospitalization [20].

### Limitations

The findings in our single tertiary care academic center MICU and CCU may not be generalizable to other centers or to patients introduced at other levels of care. Of note, data collected spanned the course of hospitalization, and may not be specific to the ICU setting either. By nature of a retrospective chart review, the relationships we found in this study are correlations that cannot imply any directional causation. Furthermore, as a chart review, our data collection was limited by what was documented in the electronic medical record. For example, presence and timing of palliative care consultation was measured based on the order placement in the electronic medical record, which may not be an accurate reflection of actual timing. Furthermore, only principal diagnosis for each patient was collected, data on the nature of illness, underlying medical problems, and reason for transfer was not included, limiting generalizability of our results. In addition, data regarding MICU and CCU patients who were not transfers from outside hospitals was not available. Thus, comparisons between our cohort and those patients who were not outside transfers cannot be made. Further studies are needed to validate our findings and define the value of palliative care involvement in critically ill patients transferred to academic medical centers.

## Conclusion

There is a high mortality rate associated with patients transferred from community hospitals to academic medical centers, yet there was a low rate of palliative care consultation in this cohort. Furthermore, there was a delay in palliative care consultation, with the majority of consults placed over 7 days into admission. This represents a resource-intensive population with which increased, rapid palliative care involvement has room for improving optimization of resources and patient outcomes in ICUs.
